# Blood pressure control for diabetic retinopathy

**DOI:** 10.1590/1516-3180.20151333T1

**Published:** 2014-12-19

**Authors:** Diana V. Do, Xue Wang, Satyanarayana S. Vedula, Michael Marrone, Gina Sleilati, Barbara S. Hawkins, Robert N. Frank

## Abstract

**BACKGROUND:**

Diabetic retinopathy is a common complication of diabetes and a leading cause of visual impairment and blindness. Research has established the importance of blood glucose control to prevent development and progression of the ocular complications of diabetes. Simultaneous blood pressure control has been advocated for the same purpose, but findings reported from individual studies have supported varying conclusions regarding the ocular benefit of interventions on blood pressure.

**OBJECTIVES:**

The primary aim of this review was to summarize the existing evidence regarding the effect of interventions to control or reduce blood pressure levels among diabetics on incidence and progression of diabetic retinopathy, preservation of visual acuity, adverse events, quality of life, and costs. A secondary aim was to compare classes of anti-hypertensive medications with respect to the same outcomes.

**METHODS:**

*Search methods:* We searched a number of electronic databases including CENTRAL as well as ongoing trial registries. We last searched the electronic databases on 25 April 2014. We also reviewed reference lists of review articles and trial reports selected for inclusion. In addition, we contacted investigators of trials with potentially pertinent data.

*Selection criteria:* We included in this review randomized controlled trials (RCTs) in which either type 1 or type 2 diabetic participants, with or without hypertension, were assigned randomly to intense versus less intense blood pressure control, to blood pressure control versus usual care or no intervention on blood pressure, or to different classes of anti-hypertensive agents versus placebo.

*Data collection and analysis:* Pairs of review authors independently reviewed titles and abstracts from electronic and manual searches and the full text of any document that appeared to be relevant. We assessed included trials independently for risk of bias with respect to outcomes reported in this review. We extracted data regarding trial characteristics, incidence and progression of retinopathy, visual acuity, quality of life, and cost-effectiveness at annual intervals after study entry whenever provided in published reports and other documents available from included trials.

**MAIN RESULTS:**

We included 15 RCTs, conducted primarily in North America and Europe, that had enrolled 4157 type 1 and 9512 type 2 diabetic participants, ranging from 16 to 2130 participants in individual trials. In 10 of the 15 RCTs, one group of participants was assigned to one or more anti-hypertensive agents and the control group received placebo. In three trials, intense blood pressure control was compared to less intense blood pressure control. In the remaining two trials, blood pressure control was compared with usual care. Five of the 15 trials enrolled type 1 diabetics, and 10 trials enrolled type 2 diabetics. Six trials were sponsored entirely by pharmaceutical companies, seven trials received partial support from pharmaceutical companies, and two studies received support from government-sponsored grants and institutional support.

Study designs, populations, interventions, and lengths of follow-up (range one to nine years) varied among the included trials. Overall, the quality of the evidence for individual outcomes was low to moderate. For the primary outcomes, incidence and progression of retinopathy, the quality of evidence was downgraded due to inconsistency and imprecision of estimates from individual studies and differing characteristics of participants.

For primary outcomes among type 1 diabetics, one of the five trials reported incidence of retinopathy and one trial reported progression of retinopathy after 4 to 5 years of treatment and follow-up; four of the five trials reported a combined outcome of incidence and progression over the same time interval. Among type 2 diabetics, 5 of the 10 trials reported incidence of diabetic retinopathy and 3 trials reported progression of retinopathy; one of the 10 trials reported a combined outcome of incidence and progression during a 4- to 5-year follow-up period. One trial in which type 2 diabetics participated had reported no primary (or secondary) outcome targeted for this review.

The evidence from these trials supported a benefit of more intensive blood pressure control intervention with respect to 4- to 5-year incidence of diabetic retinopathy (estimated risk ratio (RR) 0.80; 95% confidence interval (CI) 0.71 to 0.92) and the combined outcome of incidence and progression (estimated RR 0.78; 95% CI 0.63 to 0.97). The available evidence provided less support for a benefit with respect to 4- to 5-year progression of diabetic retinopathy (point estimate was closer to 1 than point estimates for incidence and combined incidence and progression, and the CI overlapped 1; estimated RR 0.88; 95% CI 0.73 to 1.05). The available evidence regarding progression to proliferative diabetic retinopathy or clinically significant macular edema or moderate to severe loss of best-corrected visual acuity did not support a benefit of intervention on blood pressure: estimated RRs and 95% CIs 0.95 (0.83 to 1.09) and 1.06 (0.85 to 1.33), respectively, after 4 to 5 years of follow-up. Findings within subgroups of trial participants (type 1 and type 2 diabetics; participants with normal blood pressure levels at baseline and those with elevated levels) were similar to overall findings.

The adverse event reported most often (7 of 15 trials) was death, yielding an estimated RR 0.86 (95% CI 0.64 to 1.14). Hypotension was reported from three trials; the estimated RR was 2.08 (95% CI 1.68 to 2.57). Other adverse ocular events were reported from single trials.

**AUTHORS’ CONCLUSIONS:**

Hypertension is a well-known risk factor for several chronic conditions in which lowering blood pressure has proven to be beneficial. The available evidence supports a beneficial effect of intervention to reduce blood pressure with respect to preventing diabetic retinopathy for up to 4 to 5 years. However, the lack of evidence to support such intervention to slow progression of diabetic retinopathy or to prevent other outcomes considered in this review, along with the relatively modest support for the beneficial effect on incidence, weakens the conclusion regarding an overall benefit of intervening on blood pressure solely to prevent diabetic retinopathy.

**The abstract of this review is available from:** http://onlinelibrary.wiley.com/doi/10.1002/14651858.CD006127.pub2/abstract.
